# Mode locking suppression in a magneto-optical gyro

**DOI:** 10.1038/s41598-020-76331-8

**Published:** 2020-11-10

**Authors:** Alexander M. Merzlikin, Roman S. Puzko

**Affiliations:** 1 Institute for Theoretical and Applied Electromagnetics, Russian Academy of Sciences, 13 Izhorskaya st., Moscow, 125412 Russia; 2 Dukhov Automatics Research Institute (VNIIA), 22 Sushchevskaya st., Moscow, 127055 Russia

**Keywords:** Photonic devices, Microresonators, Magneto-optics

## Abstract

Integrated ring laser gyroscopes are perfect candidates for small-sized and high-performance gyroscopes. However, the performance of the ring laser gyroscope (RLG) near zero angular velocity is fundamentally restricted by the mode locking effect. In the paper the magneto-optical ring resonator is studied as a sensitive element of the integrated RLG. The counter-propagating waves are generated at the same frequency for resonator at rest and are spatially split. It is shown that the spatial splitting of modes in such a resonator drastically suppresses the mode locking problem even at the near zero angular velocity.

## Introduction

The area of gyroscopes application covers electronics, vehicles, robotics and medical instruments. The miniaturization trend of electronic and mechanical devices requires compact yet precise gyroscopes. The development and production of the miniature gyroscope with a resolution less than $1^{\circ }/{\text{ h }}$ are important technological tasks that stimulate research efforts^1^. There are three well-known technological approaches to the problem^1^: the improvement of micro-electro-mechanical gyroscopes (MEMS gyros)^2^, the development of compact nuclear magnetic resonance gyroscopes (NMR gyros)^3–5^, and the miniaturization of optical gyroscopes by means of integrated optics technology (on-chip optical gyros)^6–8^. The MEMS gyroscopes have almost reached their performance limits, and the miniature NMR gyroscopes are still being developed. At the same time, integrated optics has a great potential as the platform for development of high-precision small-sized gyros: recently developed high-quality optical micro-resonators allow production of highly sensitive systems based on the Sagnac effect^1,7,9^.

**Figure 1:**
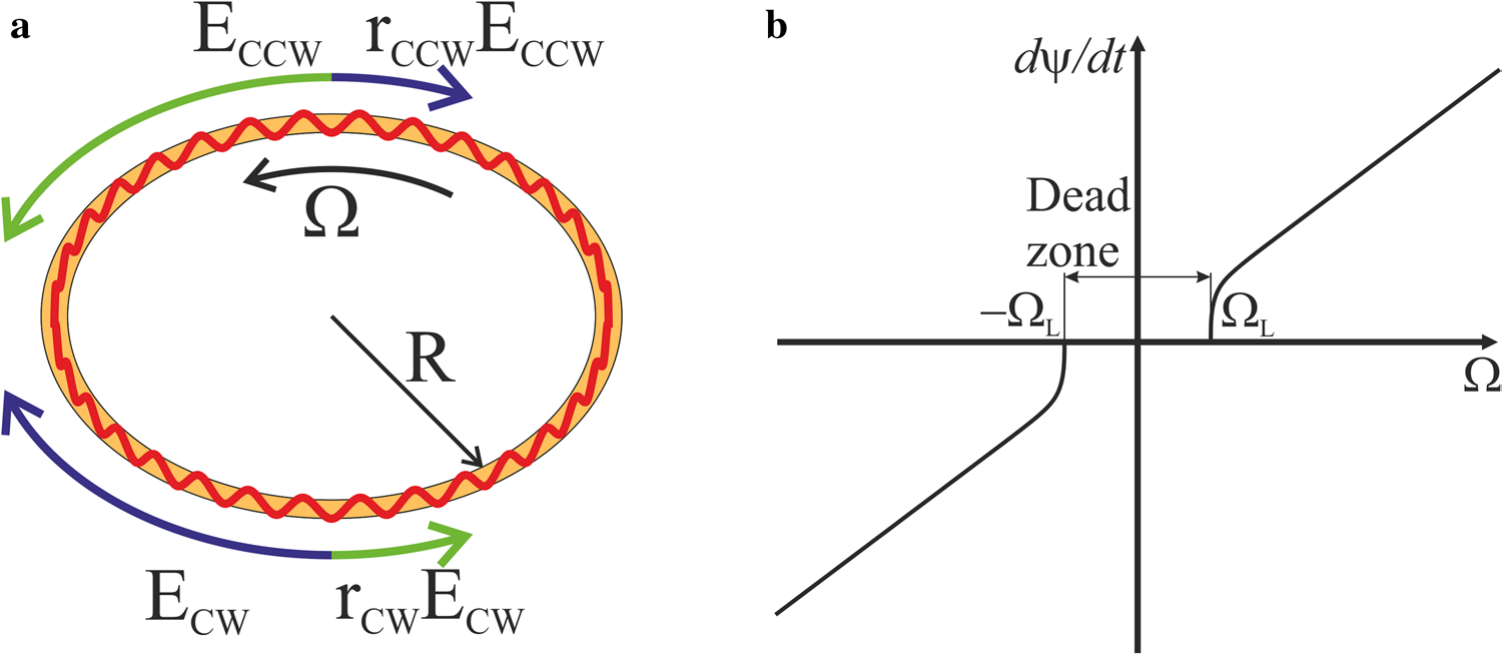
(**a**) The propagation of CW and CCW waves in a ring resonator. $E_{CW}$ and $E_{CCW}$ stand for the wave amplitudes, $r_{CW}$ and $r_{CCW}$ are the backscattering coefficients per resonator pass. (**b**) The typical dependency of gyroscope signal on the angular velocity $\Omega$.

The integrated ring laser gyroscope (IRLG) is a promising on-chip optical gyroscope with theoretically predicted high performance^1,8^. The sensing element of such gyroscope is an integrated ring laser with electrical^10–12^ or optical pumping^13^. IRLG operation principle is similar to the one of a full-sized ring laser gyroscope (RLG): two counter-propagating waves (clockwise (CW) and counterclockwise (CCW)) are generated in the laser resonator (see Fig. 1a). The frequencies of the waves are different due to the Sagnac effect and are detected in the device.

Previously, various designs of IRLG were proposed^1^. However, the laser gyroscope of any design faces the inevitable problem of mode locking. The mode locking is the main problem which restricts the miniaturization of RLG^1^. The interaction between counter-propagating waves results in their locking if the angular velocity of the gyroscope is below certain critical value. The counter-propagating waves acquire the same frequency in that case. As a result the dead zone of device sensitivity is formed in the range from $-\Omega _L$ to $\Omega _L$ ($\Omega _L$ is the maximum angular velocity of the dead zone) near zero angular velocity (see Fig. 1b).

The mode locking in RLG was actively discussed in literature^14–16^. The interaction between counter-propagating waves (that is caused by the backscattering^14^) leads to the energy exchange between CW and CCW resonator waves (Fig. 1a). The linear backscattering occurs on the system imperfections: surface roughness or volume inhomogenities. The nonlinear backscattering is the interaction between the waves caused by gain medium^17^. Due to the dependency of gain on the radiation intensity the spatial and temporal variations of field result in gain modulation^18^. The gain modulation acts as the grating which scatters the radiation.

To avoid the mode locking effect in a full-sized laser gyroscope the additional constant/dynamic dithering is applied. The dithering shifts the angular velocity “experienced” by gyroscope outside of the dead zone. A number of dithering methods were proposed, such as the physical rotation of the gyroscope^19–21^ or the introduction of non-reciprocal optical elements^22–25^.

The mechanical dithering is provided by the physical rotation of gyroscope. However, that requires the rotating system, which influences the noise characteristics of the gyro. In particular, the angular random walk (ARW) increases. The magneto-optical dithering is an alternative to the mechanical dithering. Magneto-optical media is used in full-sized gyroscopes as non-reciprocal optical element, providing the phase shift of the counter-propagating waves in the gyroscope. The non-reciprocal properties of wave propagation in magneto-optical elements allow to control the difference between the frequencies/phases of the counter-propagating waves, i.e., it is possible to control the mode locking.

The mode locking is suppressed in the full-sized Zeeman gyroscopes^26,27^ by the frequency splitting of the counter-propagating waves. The sensitive element of the gyroscope is a ring laser with a non-planar resonator, which is placed in a magnetic field. The modes of non-planar ring resonator are left-handed and right-handed circularly polarized waves. Due to the Zeeman effect the gain curve acquires Zeeman splitting in a magnetic field, which results in the generation of waves with the different frequencies inside the resonator.

Notably, the dithering switches the sensing frequency of the gyro away from the dead zone rather than suppresses mode locking. The giant mode locking in IRLG nullifies the advantages of dithering approaches. The mode locking is suppressed by reduction of waves interaction (e.g., by use of high quality mirrors or modes with different polarization). The existing manufacturing technology allows the production of high quality nanostructures with subnanometer scale roughness^28,29^. The fine structure of the resonator results in the suppression of light scattering. Although the linear backscattering can be made negligible in such resonators, the mode locking occurs due to the nonlinearity of the gain medium. The nonlinearity is the inherent property of gain medium and manifests itself in both the gaseous and the integrated RLG. However, the gain is significantly higher in the IRLG^30^, which results in higher nonlinearity and increased mode locking effect. Thus, the gain nonlinearity is the predominant cause of mode locking in the IRLG. In this paper we propose the promising way to suppress the mode locking.

Recent advances in photonics allow the use of materials with peculiar optical properties, which opens up great opportunities for suppression of mode locking. In this paper, it is proposed to use a magneto-optical resonator in an integrated optical gyroscope. The modes splitting in a cavity made entirely of a magneto-optical material is investigated.

## Results

The “Results” section is organized as follows. In the first part the principle of mode locking suppression by spatial splitting of modes is given. In the next section, the certain resonator design and its modes are considered. Finally, the suppression of mode locking in the resonator is studied.

### Spatial splitting of modes

The operation principle of the optical gyroscope is based on the Sagnac effect: the phase difference builds up between the counter-propagating waves in the rotating ring interferometer, which depends on the angular velocity of rotation. The difference $\Delta \nu$ between the counter-propagating waves in RLG is proportional to the angular velocity $\Omega$ of the device

1 $$\begin{aligned} \Delta \nu =S\Omega =\frac{4a}{p\lambda }\Omega \end{aligned}$$

where *S* is the scale factor of Sagnac effect for the gyro, *a* and *p* are the area and the perimeter of the light path in the gyro, $\lambda$ is the wavelength. The value of $\Delta \nu$ is measured in the device.

However, the backscattering effects present in the RLG, which influence the measurements. Namely, the total phase shift $\psi$ between the counter-propagating waves is approximately satisfies the following differential equation^31^:

2 $$\begin{aligned} \frac{d\psi }{dt}=2\pi S\Omega +\kappa sin\left( \psi +\eta \right) \end{aligned}$$

where $\kappa$ is the backscatter coefficient, measured in the units of frequency and taking into account all backscattering effects in the resonator, $\eta$ is the constant phase due to the backscattering.

The Eq. (2) is phenomenological one, i.e. the nature of backscattering is not specified. The coefficient $\kappa$ considers all the effects leading to modes interaction. In the case of large angular velocity ($\kappa \ll 2\pi S\Omega$) the Eq. (2) does not have a stationary solution and the frequency shift depends linearly on the angular velocity. In the case of small angular velocity ($\kappa \sim 2\pi S\Omega$ or $\kappa \gg 2\pi S\Omega$) the differential equation has a stationary solution, and the difference between the frequencies of counter-propagating waves becomes zero, i.e. mode locking occurs.

The backscattering coefficient $\kappa$ describes both the linear and the nonlinear scattering, which occurs in the sensing element of gyro—the ring resonator. The improvement of manufacturing process suppress the linear scattering. At the same time the nonlinear scattering is the inherent property of the gain medium. The specific optical resonator is considered in order to suppress the nonlinear backsacttering.

**Figure 2:**
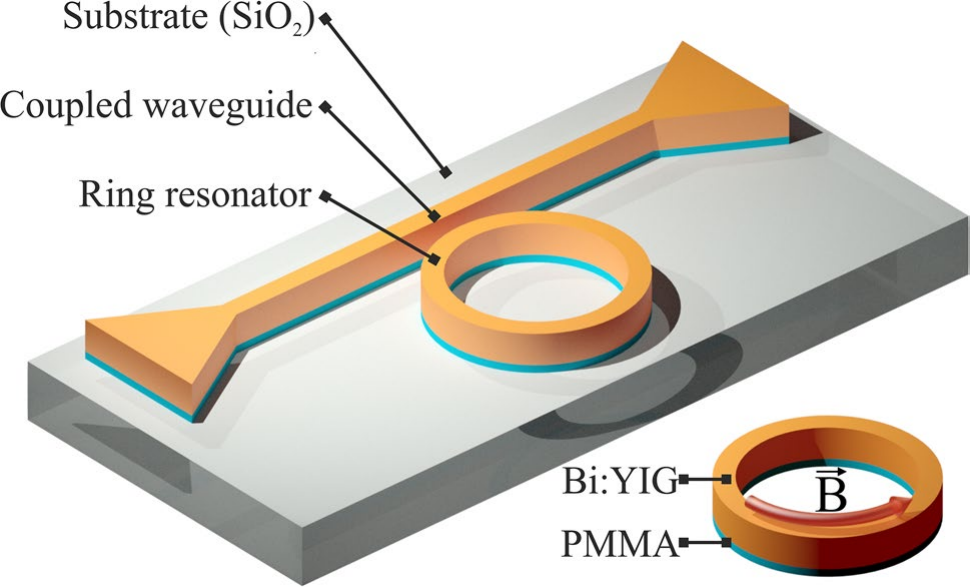
The scheme of the integrated magneto-optical gyroscope.

The resonator under discussion is composed of a layer of magneto-optical material placed on a gain layer (see Fig. 2). The gain layer of the ring resonator is made of a dielectric with quantum dots. These quantum dots act as the active laser medium.

A magnetic field is applied to the resonator, oriented along the circumference of the resonator. The additional wired system can be used in order to create such field. The read-out system is connected to the resonator by means of a coupling waveguide. This technology is widely used in a various integrated optical devices including passive gyroscopes.

The dielectric tensor of the magneto-optical material has the form

3 $$\begin{aligned} \hat{\varepsilon }_{MO}=\left( \begin{matrix} \varepsilon &{}&{} ig &{}&{} 0\\ -ig &{}&{} \varepsilon &{}&{} 0\\ 0 &{}&{} 0 &{}&{} \varepsilon \end{matrix}\right) \end{aligned}$$

where the off-diagonal element *g* depends on the applied magnetic field. The propagation constants of counter-propagating waves are different in such a medium: the difference is $\Delta {k}\propto {g}$ in the homogeneous magneto-optical medium. In the single-mode waveguide there are two counter-propagating waves, which have different polarizations and propagation constants. Notably, the waves are approximately elliptically polarized, which significantly affects interference.

The counter-propagating waves with the propagation constants $\beta _{CW}$ and $\beta _{CCW}$ are the resonant modes of a ring resonator with a certain length *L* when the following conditions are satisfied

4 $$\begin{aligned} \beta _{CW}L&=2\pi {N}, \end{aligned}$$

5 $$\begin{aligned} \beta _{CCW}L=2\pi \left( N+1\right) , N\in {\mathbb {Z}} \end{aligned}$$

Since the propagation constants differ, the spatial periodicities of the modes field distributions along the resonator differ. That leads to a decrease in the modes interaction caused by nonlinear backscattering.

### Magneto-optical gyro design

The considered configuration of the resonator—the magneto-optical ring—is depicted in Fig. 2. The magneto-optical waveguide is made of Bi:YIG and PMMA layers. The width and height of the Bi:YIG layer are 220 nm and 200 nm, correspondingly. The Bi:YIG layer is placed on a 50 nm thick PMMA layer. The structure is placed on a glass substrate. It is assumed that the quantum dots necessary for modes generation are placed inside the PMMA layer.

**Figure 3:**
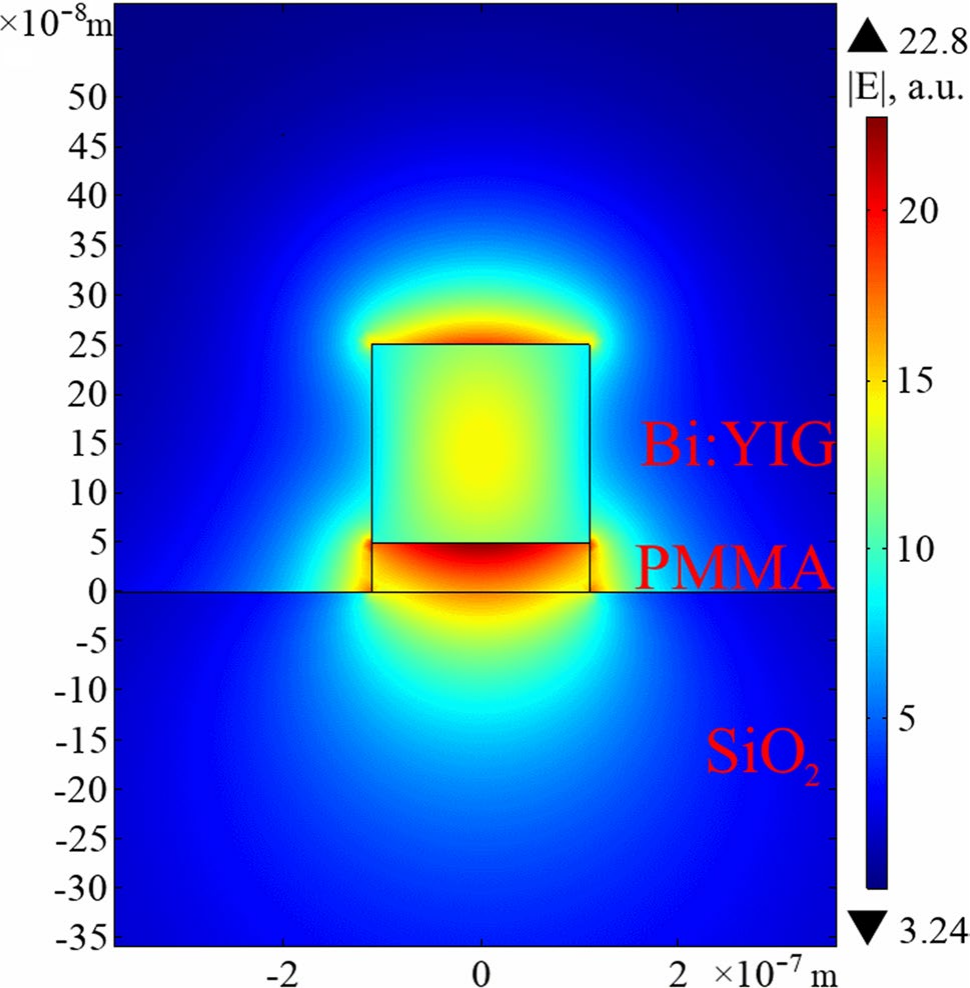
The distribution of the electric field modulus at the cross section of the considered magneto-optical waveguide. The width of the waveguide is 220 nm, the height of the Bi:YIG layer is 200 nm, the height of PMMA layer is 50 nm. The wavelength is 800 nm.

The considered structure is a single-mode waveguide at the wavelength 800 nm. The refractive indices of the PMMA layer and glass are taken to be $n_{PMMA}=1.4968$ and $n_{Glass}=1.4533$, correspondingly. The dielectric permittivity tensor of Bi:YIG can be written in the form (3), where the diagonal element of the tensor $\varepsilon =5.56$^32^. The off-diagonal element *g* can be as high as $1.98 \times {10^{-3}}$ at the saturation. The magnetizing field $\vec {B}$ is oriented along the waveguide circumference (see Fig. 2). Two counter-propagating modes have different elliptical polarizations and different propagation constants. Nevertheless, the difference between propagation constants quite small and field distributions at waveguide cross section are similar for both modes. In Fig. 3 one can see the distribution of the electric field of the waveguide mode at the cross section of considered resonator.

**Figure 4:**
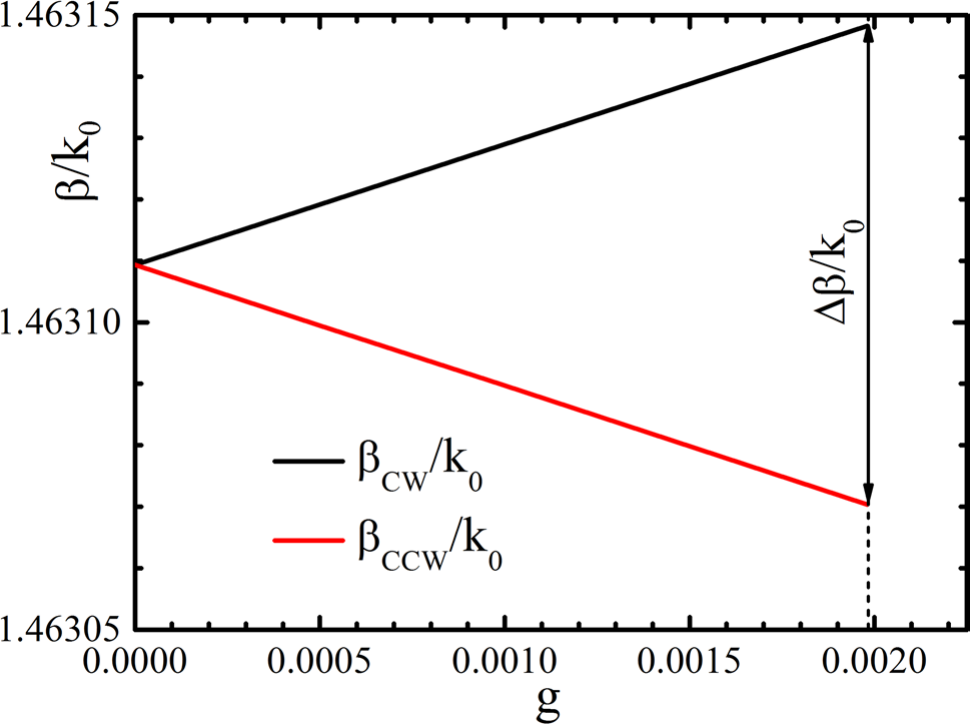
The dependence of the propagation constants of the counter-propagating waveguide modes (CW and CCW) on the off-diagonal element of the dielectric permittivity tensor. The waveguide geometry is the same as on Fig. 3.

In the Bi:YIG the value of *g* can be controlled by the applied magnetic field, as in the wide range of field amplitude (until the saturation takes place) *g* is proportional to the value of applied magnetic field. The dependences of the modes propagation constants on *g* are shown in Fig. 4. The dependences are approximately linear. The saturation value $g=1.98\times {10^{-3}}$ corresponds to the difference $\Delta {\beta }=7.8\times {10^{-5}}k_0$ between the counter-propagating modes, where $k_0$ is the wave vector in free space. In this case, the length of the resonator required to fulfill conditions (4) and (5) is $L=2\pi /\Delta {\beta }\approx 10.4$ mm (the diameter of the resonator ring is 3.3 mm).

### Mode locking and sensitivity

Now let us consider in detail the influence of spatial splitting on the mode locking effect. The phase shift $\psi$ between the counter-propagating modes with eigenfrequencies $\omega _{CW}$ and $\omega _{CCW}$ is described by following differential equation^14^

6 $$\begin{aligned} \frac{d\psi }{dt}=(\omega _{CCW}-\omega _{CW})+\kappa sin\left( \psi +\eta \right) \end{aligned}$$

where $\kappa$ is the backscattering coefficient. The backscattering is caused by two main processes: linear backscattering due to the waveguide imperfections and nonlinear backscattering caused by the nonlinearity of gain medium. To estimate the effectiveness of mode locking suppression in magneto-optical waveguide the contributions of both effects to the mode locking should be evaluated.

First, let us consider the nonlinear backscattering. Both counter-propagating waves are amplified by the same gain medium. The gain in the resonator is modulated by the electric field due to gain saturation. In result, modulation of the gain medium leads to the modes coupling.

The saturation effect of the gain arises under the influence of the radiation propagating in the waveguide. If the intensity $I_r$ of propagating waves is much lower than the saturation intensity $I_s$, the correction to the gain (and corresponding dielectric permittivity) proportional to the field intensity^18^

7 $$\begin{aligned} \delta \varepsilon \propto \frac{I_r}{I_s}\propto \left| {\text {Re}}\left[ \vec {E}\left( x,y,z\right) \right] \right| ^2 \end{aligned}$$

In the framework of coupled mode theory (see “Methods” section for details) the first order correction to $\delta \varepsilon$ is

8 $$\begin{aligned} \delta \varepsilon \propto \left| E^0_{CW}\right| ^2+\left| E^0_{CCW}\right| ^2 +2\left| E^0_{CW}E^0_{CCW}\right| {\text {cos}}\left( (\beta _{CCW}-\beta _{CW})z +\psi (t)\right) \end{aligned}$$

where $E^0_{CW}=\left| E^0_{CW}\right| e^{i\phi _{CW}}$ and $E^0_{CCW}=\left| E^0_{CCW}\right| e^{i\phi _{CCW}}$ are the amplitudes of the counter-propagating waves. The first and second terms of (8) do not depend on the phases or the spatial distribution of the field along the waveguide. Therefore, they only lead to renormalization of the gain value. The last term depends on the phase difference between waves. Thus, the correction to the medium parameters depends on interference between counter-propagating waves, leading to gain modulation and mode interaction. However, the wave coupling after the propagation through entire resonator circumference is absent due to the suppression of waves interference [see (4) and (5)].

Thus, the nonlinear backscattering does not lead to wave coupling in the first order of perturbation theory, which results in the reduction of counter-propagating waves coupling and the mode locking suppression.

Let us now consider the effect of the linear backscattering on mode locking. Light backscattering due to the roughness of the waveguide leads to an additional coupling between the modes, characterized by the scattering coefficients $r_{CW}$ and $r_{CCW}$ (see Fig. 1a). The Q factor of the resonator with the roughness about 1 nm can be as high as $10^{6}$–$10^{8}$ (the value is in agree with the existing technological processes), i.e., the scattering coefficients per resonator turn are $r_{CW/CCW}\sim 10^{-2}$. However, the linear scattering by the waveguide roughness is uncorrelated process. That decreases the effective scattering coefficients $r_{eff}\sim r_{CW/CCW}\sqrt{L/L_c}$^33^. Here $L_c$ is a roughness correlation length, and *L* is the resonator length. The gyroscope dead band width is $\Omega _L=\kappa /(2\pi S)\approx cr_{eff}/(2\pi SL)\,\sim 10^4 {^{\circ }/{\text {h}}}$ for a gyroscope with a resonator length $L\approx 10.4\ \hbox {mm}$. This value corresponds to the magnitude of the mode locking in a full-sized gyro.

Thus, the effect of mode coupling caused by the gain medium is reduced in the considered magneto-optical resonator. On the other hand, the operation principle of the gyroscope remains the same, and its limiting sensitivity corresponds to the level of shot noise, which has a value of $\sim 10^{-2} {^{\circ }/{\text {h}}}$ for a gyroscope of a similar configuration^1^.

## Discussion

The on-chip optical laser gyroscope is a promising miniature angular velocity sensor. The implementation of the device is based on the gain medium with a high value of gain. However, high gain is accompanied by the strong mode interaction in the laser resonator and, consequently, the giant mode locking. The mode locking is crucial problem for the IRLG development.

The dithering approaches (mechanical dithering, non-reciprocal optical elements) are used in full-sized RLGs to avoid the mode locking. These approaches based on the shift of the dead zone and as a rule slightly worsen the gyroscope precision. At the same time dithering does not influence the cause of the mode locking effect—waves backscattering. The backscattering by waveguide roughness can be diminished by appropriate production technology. However, the main reason for the mode locking in the IRLG is the backscattering due to the nonlinearity of the gain medium.

In the paper we proposed the magneto-optical resonator in order to suppress the influence of gain nonlinearity on the mode interaction. It is proposed to use the magneto-optical ring waveguide made of Bi:YIG as a resonator of the gyroscope. The magnetizing field leads to the magneto-optical split of the counter-propagating waves.

In contrast to the magneto-optical full-sized RLGs the mode locking problem is solved by spatial splitting of counter-propagating modes rather than by frequency splitting. The frequencies of the sensing modes are the same for the gyro at rest. However, their propagation constants and polarizations are different. It is shown in the paper that the mode coupling is suppressed due to the displacement of the modes antinodes, which is confirmed by coupled mode theory. Thus, the mode locking is suppressed by reducing of the nonlinear backscattering in the gain medium. At the same time the existing technological processes allows the production of the proposed resonator with high accuracy, which means low linear backscattering as well. In this case, the maximum theoretical sensitivity of the gyroscope $<1{^{{\circ }}}/{\text{ h }}$.

## Methods

### Resonator modes

The computations of the propagation constants and mode shapes at waveguide cross sections were made in COMSOL Multiphysics.

### Nonlinear backscattering

Let us consider the coupling between counter-propagating waves caused by the nonlinear backscattering. Both CW and CCW waves are amplified by the same gain medium. The gain value in the resonator is modulated by the propagating waves. As a result, modulation of the gain medium leads to the waves coupling.

Let us consider the field inside the waveguide as a superposition of CW and CCW fields

9 $$\begin{aligned} \vec {E}\left( x,y,z\right) =U_{CW}(x,y)A_{CW}(z,t)\vec {e}_{CW} +U_{CCW}(x,y)A_{CCW}(z,t)\vec {e}_{CCW} \end{aligned}$$

where *z* is the coordinate along the resonator ring, (*x*, *y*) is the waveguide cross section plane, $\vec {e}_{CW}$ and $\vec {e}_{CCW}$ are the polarization vectors of counter-propagating waves, $U_{CW/CCW}(x,y)$ is the field distribution at the cross section of the waveguide, $A_{CW}(z,t)$ and $A_{CCW}(z,t)$ are the amplitudes of the counter-propagating waves. In an ideal waveguide without gain/absorption

10 $$\begin{aligned} A_{CW}(z,t)&=E^0_{CW}e^{-i\beta _{CW}z-i\omega _{CW}t} \end{aligned}$$

11 $$\begin{aligned} A_{CCW}(z,t)&=E^0_{CCW}e^{i\beta _{CCW}z-i\omega _{CCW}t} \end{aligned}$$

The field $\vec {E}\left( x,y,z\right)$ satisfies the wave equation

12 $$\begin{aligned} \Delta \vec {E}\left( x,y,z\right) -\frac{\hat{\varepsilon } \left( x,y\right) }{c^2}\frac{\partial ^2\vec {E}\left( x,y,z\right) }{\partial t^2}=0 \end{aligned}$$

where the tensor $\hat{\varepsilon }$ inside the magneto-optical medium has the form (3). The dielectric tensor $\hat{\varepsilon }$ is a diagonal inside the PMMA layer and in the glass. To account for gain, we add to the dielectric permittivity of gain layer (PMMA) a negative imaginary part.

In the framework of the perturbation theory, the gain modulation leads to perturbations of field and complex dielectric permittivity

13 $$\begin{aligned}&U_{CW/CCW}\left( x,y\right) \rightarrow U_{CW/CCW}\left( x,y\right) +\delta U_{CW/CCW}\left( x,y\right) \end{aligned}$$

14 $$\begin{aligned}&E^0_{CW/CCW}\rightarrow E_{CW/CCW}\left( z,t\right) \end{aligned}$$

15 $$\begin{aligned}&\hat{\varepsilon }\left( x,y\right) \rightarrow \hat{\varepsilon } \left( x,y\right) +\delta \varepsilon \left( x,y,z,t\right) \end{aligned}$$

where we assume that the function $E_{CW/CCW}\left( z,t\right)$ is a slowly varying function of time and coordinate, accounting for field amplification due to gain, as well as for the variations in the amplitude of the mode due to the gain modulation. In the first order of the perturbation theory, we obtain an equation for the amplitudes of the counter-propagating waves

16 $$\begin{aligned} M_{CW}e^{-i\beta _{CW}z-i\omega _{CW}t}\vec {e}_{CW}+M_{CCW} e^{i\beta _{CCW}z-i\omega _{CCW}t}\vec {e}_{CCW}=0 \end{aligned}$$

where

17 $$\begin{aligned} M_{CW}&=\left( \Delta _\perp \delta {U_{CW}}\right) E_{CW} -\beta ^2_{CW} \delta {U_{CW}}E_{CW}-2i\beta _{CW}\frac{\partial {E_{CW}}}{\partial {z}}U_{CW} +\hat{\varepsilon }\frac{\omega ^2_{CW}}{c^2} \delta {U_{CW}}E_{CW}\nonumber \\&\quad +\delta \hat{\varepsilon }\frac{\omega ^2_{CW}}{c^2}U_{CW}E_{CW} +2i\hat{\varepsilon }\frac{\omega _{CW}}{c^2} \frac{\partial {E_{CW}}}{\partial {t}}U_{CW}, \end{aligned}$$

18 $$\begin{aligned} M_{CCW}&=\left( \Delta _\perp \delta {U_{CCW}}\right) E_{CCW}-\beta ^2_{CCW} \delta {U_{CCW}}E_{CCW}+2i\beta _{CCW}\frac{\partial {E_{CCW}}}{\partial {z}}U_{CCW} +\hat{\varepsilon }\frac{\omega ^2_{CCW}}{c^2}\delta {U_{CCW}}E_{CCW}\nonumber \\&\quad +\delta \hat{\varepsilon }\frac{\omega ^2_{CCW}}{c^2}U_{CCW}E_{CCW} +2i\hat{\varepsilon }\frac{\omega _{CCW}}{c^2} \frac{\partial {E_{CCW}}}{\partial {t}}U_{CCW}, \end{aligned}$$

and $$\Delta _\perp =\frac{\partial ^2}{\partial {x}^2} +\frac{\partial ^2}{\partial {y}^2}$$.

The total contribution of the nonlinear backscattering to the mode locking after the propagation along the entire resonator circumference can be evaluated after the integration of (16) along the resonator circumference.

The mode interaction is strongly depends on the polarization vectors $\vec {e}_{CW}$ and $\vec {e}_{CCW}$. The magneto-optical resonator has the dielectric tensor (3) being placed in the magnetic field. That leads not only to the difference in the propagation constants of the modes but also to the different polarizations of the modes. Notably, the waves are elliptically polarized, which affects the interference.

Consider the change of CW amplitude due to the interaction with the CCW wave. Generally, there are two expressions which may lead to the interaction between CW and CCW amplitudes

19 $$\begin{aligned}&\vec {e}_{CW}E_{CW}\int {\delta \varepsilon (x,y,z,t)dz} \end{aligned}$$

20 $$\begin{aligned} \vec {e}_{CCW}E_{CCW}\int {\delta \varepsilon (x,y,z,t) {\text {exp}}\left( i\left( \beta _{CW}+\beta _{CCW}\right) z\right) dz} \end{aligned}$$

The first expression is proportional to the average gain modulation $\delta \varepsilon$ along the resonator circumference. Here it is important that non-zero average of $\delta \varepsilon$ does not necessary leads to the mode locking. The mode locking occurs if expression (19) depends on the amplitude or phase of CCW wave. The second expression (20) is the average gain modulation with respect to the phase difference between CW and CCW waves. As soon as it contains the amplitude of CCW wave the expression (20) leads to mode locking in the general case. However, the different mode polarization suppresses mode locking effect given by (20). i.e., the mode locking in the considered case depends only on the expression (19).

The perturbation of the CCW amplitude depends on two expressions, which are

21 $$\begin{aligned}&\vec {e}_{CCW}E_{CCW}\int {\delta \varepsilon (x,y,z,t)dz} \end{aligned}$$

22 $$\begin{aligned}&\vec {e}_{CW}E_{CW}\int {\delta \varepsilon (x,y,z,t){\text {exp}} \left( -i\left( \beta _{CW}+\beta _{CCW}\right) z\right) dz} \end{aligned}$$

The consideration of expressions (21) and (22) is analogous to the one of (19) and (20).

Now, consider (19) in detail. The saturation effect of the gain $\delta \varepsilon$ arises under the influence of the radiation propagating in the waveguide. If the intensity $I_r$ of the propagating waves is much lower than the saturation intensity $I_s$, the correction to the corresponding complex dielectric permittivity is^18^

23 $$\begin{aligned} \delta \varepsilon \propto \frac{I_r}{I_s}\propto \left| {\text {Re}} \left[ \vec {E}\left( x,y,z\right) \right] \right| ^2 \end{aligned}$$

Neglecting the slow variations of $E_{CW/CCW}(z,t)$ on *z* and *t*, one obtains

24 $$\begin{aligned} \delta \varepsilon \propto \left| {\text {Re}}\left[ \left| E^0_{CW} \right| e^{i\phi _{CW}(t) -i\beta _{CW}z}\vec {e}_{CW}+\left| E^0_{CCW}\right| e^{i\phi _{CCW}(t)+i\beta _{CCW}z} \vec {e}_{CCW}\right] \right| ^2 \end{aligned}$$

where $E^0_{CW}e^{-i\omega _{CW}t}=\left| E^0_{CW}\right| e^{i\phi _{CW}(t)}$ and $E^0_{CCW}e^{-i\omega _{CCW}t}=\left| E^0_{CCW}\right| e^{i\phi _{CCW}(t)}$ are the amplitudes of the counter-propagating waves. Notably, the polarization vectors corresponds to the left-handed and right-handed elliptical polarizations. The polarizations are generally differ from the elliptical polarizations inside the waveguide. For simplicity, we consider that the CW and CCW waves are approximately circularly polarized. The resulting expression for the gain modulation is as follows

25 $$\begin{aligned} \delta \varepsilon \propto \left| E^0_{CW}\right| ^2+\left| E^0_{CCW}\right| ^2 +2\left| E^0_{CW}E^0_{CCW}\right| {\text {cos}}\left( (\beta _{CCW} -\beta _{CW})z+\psi (t)\right) \end{aligned}$$

where $$\psi (t)=\phi _{CW}(t)+\phi _{CCW}(t)$$.

The first two terms do not depend on the spatial distribution of the field along the waveguide. Therefore, they only lead to renormalization of the gain value. However, the last term leads to the dependency of the CW wave amplitude on $\left| E^0_{CW}E^0_{CCW}\right| cos \left( \left( \beta _{CCW}-\beta _{CW}\right) z+\psi (t)\right)$ at some point *z* along the ring resonator, i.e. it leads to the waves coupling. However, the wave coupling after the propagation through entire resonator circumference is absent as the integral of the last term in (25) along the resonator circumference turns to zero in accordance with (4) and (5).

Note, that the magneto-optical properties of resonator material suppress the mode-locking in two ways. First of all, the elliptical polarization of waves nullifies the effect of modes interaction due to (20) and (22). Secondly, the spatial splitting of modes nullifies nonlinear backscattering due to the interference of the modes in (25).
